# Stress Perfusion Cardiac Magnetic Resonance in Long-Standing Non-Infarcted Chronic Coronary Syndrome with Preserved Systolic Function

**DOI:** 10.3390/diagnostics12040786

**Published:** 2022-03-23

**Authors:** Pierpaolo Palumbo, Ester Cannizzaro, Annamaria Di Cesare, Federico Bruno, Francesco Arrigoni, Alessandra Splendiani, Antonio Barile, Carlo Masciocchi, Ernesto Di Cesare

**Affiliations:** 1 Department of Diagnostic Imaging, Area of Cardiovascular and Interventional Imaging, Abruzzo Health Unit 1, Via Saragat, Località Campo di Pile, 67100 L’Aquila, Italy; estercannizzaro@hotmail.it (E.C.); arrigoni.francesco@gmail.com (F.A.); 2 SIRM Foundation, Italian Society of Medical and Interventional Radiology (SIRM), 20122 Milan, Italy; federico.bruno.1988@gmail.com; 3 Ospedale “Infermi” di Rimini, Viale Luigi Settembrini, 2, 47923 Rimini, Italy; annamariadicesare.adc@gmail.com; 4 Department of Applied Clinical Sciences and Biotechnology, University of L’Aquila, Via Vetoio 1, 67100 L’Aquila, Italy; alessandra.splendiani@univaq.it (A.S.); antonio.barile@univaq.it (A.B.); carlo.masciocchi@univaq.it (C.M.); 5 Department of Life, Health and Environmental Sciences, University of L’Aquila, Piazzale Salvatore Tommasi 1, 67100 L’Aquila, Italy

**Keywords:** stress perfusion CMR, ischemia, CAD, CCS, long-standing CCS, CCTA, strain, CAD extension, heart failure

## Abstract

(1) Background: The impact of imaging-derived ischemia is still under debate and the role of stress perfusion cardiac magnetic resonance (spCMR) in non-high-risk patient still needs to be clarified. The aim of this study was to evaluate the impact of spCMR in a case series of stable long-standing chronic coronary syndrome (CCS) patients with ischemia and no other risk factor. (2) Methods: This is a historical prospective study including 35 patients with history of long-standing CCS who underwent coronary CT angiography (CCTA) and additional adenosine spCMR. Clinical and imaging findings were included in the analysis. Primary outcomes were HF (heart failure) and all major cardiac events (MACE) including death from cardiovascular causes, myocardial infarction, or hospitalization for unstable angina, or resuscitated cardiac arrest. (3) Results: Mean follow-up was 3.7 years (IQR: from 1 to 6). Mean ejection fraction was 61 ± 8%. Twelve patients (31%) referred primary outcomes. Probability of experiencing primary outcomes based on symptoms was 62% and increased to 67% and 91% when multivessel disease and ischemia, respectively, were considered. Higher ischemic burden was predictive of disease progression (OR: 1.59, 95%CI: 1.18–2.14; *p*-value = 0.002). spCMR model resulted non inferior to the model comprising all variables (4) Conclusions: In vivo spCMR-modeling including perfusion and strain anomalies could represent a powerful tool in long-standing CCS, even when conventional imaging predictors are missing.

## 1. Introduction

Chronic coronary syndrome (CCS) includes a wide spectrum of clinical scenarios involving patients with known or suspected coronary artery disease (CAD) [1].

CAD is a chronic and often progressive disease, and CCS definition has recently been introduced to differentiate a clinical stable presentation from an acute presentation (acute coronary syndrome or ACS). Different outcomes, however, can occur due to the dynamic nature of CAD, and ACS can also destabilize a long-standing (i.e., more than one year after initial diagnosis or revascularization) apparently stable clinical scenario. Therefore, correct risk stratification and adequate clinical management of CCS is essential to reduce the risk of major cardiac events [1].

Advanced cardiac imaging plays a primary role in the assessment of heart disease, both in ischemic and non-ischemic cardiomyopathy [2,3,4,5,6,7].

Based on the Bayesian probability of CAD, anatomical strategy with coronary CT angiography (CCTA) finds a prevalent role in patients with a low likelihood to have CAD [1,8,9]. From the PROMISE study, the high ability of CCTA to identify a low-risk group corresponds to an event rate of 0.9% vs. 2.1% observed in patients managed with conventional stress testing (over a two-year period of observation) [10,11,12]. Conversely, CCTA finds only a marginal role in long-standing CCS for the lack of functional information related to ischemia [1].

On the other hand, functional tests imaging for ischemia detection finds a primary role in patients with an intermediate-to-high probability of CAD and in patients with long-standing CCS, both in symptomatic or asymptomatic patients, given the risk for complications also in an otherwise asymptomatic patient. However, the impact of non-invasive imaging strategies for ischemia detection to guide initial coronary revascularization and improve long-term outcomes is still under debate [13].

Recently, the ISCHEMIA trial caused major controversy reporting no substantial benefit of ischemia testing in CAD prognostication and patient stratification, especially in the early time-window of observation and in patient with a good systolic performance, proving no superiority of an initial invasive vs. conventional medical treatment when moderate-to-severe ischemia is detected [14,15].

From ISCHEMIA, different questions arise regarding which ischemia test should be performed and which subcategories can benefit from a more aggressive treatment [16]. In this regard, from the extended STICHES, revascularization strategies plus medical therapies seem more effective than medical therapy alone in treatment of patients with reduced left ventricular ejection fraction (LVEF), thus suggesting a real benefit to coronary revascularization in patients with both ischemia and reduced EF heart failure (HFrEF) [17,18].

During the latest years, stress perfusion cardiac magnetic resonance (spCMR) showed a relevant impact in CAD stratification in many trial and registry studies [16,19,20,21,22,23,24,25]. Differently from other imaging techniques, spCMR offers a holistic approach to the heart patient through the simultaneous evaluation of the triad systolic function–perfusion abnormalities–tissue characterization [26].

CMR showed high accuracy in ischemia detection and is currently considered the gold standard for cardiac volume and systolic function evaluation [27,28]. Moreover, late gadolinium enhancement (LGE) as an imaging marker of myocardial scarring is a well-known predictor of all-cause mortality from different studies including ischemic and non-ischemic cardiomyopathies [29,30,31,32,33].

Given these discrepancies, spCMR may impact more efficiently than other conventional ischemia tests (largely involved in ISCHEMIA), although its clinical utility should be defined especially if conventional outcome predictors are missing.

The purpose of our study was to assess the impact of spCMR findings in a case series of apparently clinically stable long-standing CAD patients with preserved EF and no previous infarction or signs of HF during a long-term follow-up.

## 2. Materials and Methods

This study was carried out after the approval of our university’s Internal Review Board.

This is a retrospective assessment of prospectively followed-up patients (historical prospective/cohort study).

We screened our database to identify patients referred to our institution for a history of long-standing CCS who had been re-submitted to CCTA (to identify unprotected CAD) and additional adenosine spCMR in a short time interval (less than 6 months) for a comprehensive evaluation and were deemed able to complete a long-term follow-up.

Long-standing CCS was defined in accordance with the latest ESC guidelines (i.e., more than one year after initial diagnosis and medical treatment or revascularization) [1].

We identified and analyzed 87 patients. Those with a history of myocardial infarction (MI) were excluded. 23 participants reported previous ACS or showed ischemic-type myocardial scarring, and therefore were excluded. Another 29 patients were excluded for the following: (a) time interval between CCTA and stress CMR examination longer than 1 year; (b) clinical condition not specifically attributable to CAD for concomitant morbidities; and (c) moderate to severe systolic dysfunction.

In the end, 35 patients matched our inclusion criteria. Eligible participants were recalled for an on-site interview performed by two specialists. All information reported within the radiology information system (RIS) was also collected.

Cardiovascular symptoms (i.e., angina and/or ischemic equivalent as dyspnea) and CAD extension (i.e., a single or multivessel disease—2 or 3 major epicardial vessel) were collected.

A healthy control group was also recruited. A healthy control group was defined for: (i). preserved EF; (ii). absence of clinical history of CAD, myocardial injury and/or systemic disease; (iii). no cardiovascular symptoms or risk factor; (iv). absence of signs of structural heart disease or LGE (23 participants; 12 males, 44 ± 9 years). The healthy controls were recruited among people referred to our center for echocardiographic suspicion of cardiomyopathy but not confirmed with CMR.

### 2.1. Cardiac Magnetic Resonance Imaging Protocol

Stress CMR protocol included assessment of cardiac function, ischemia testing, and LGE imaging to exclude myocardial scarring. Resting perfusion was not included in our standard protocol.

For the assessment of LV volumes/systolic function steady-state free precession cine images (echo time/reception time 1.5/3.0 ms, flip angle 60°) were acquired on short-axis (slice thickness 8 mm, spacing 0 mm) and radial long-axis views (ten slices covering the entire circumference of the ventricle, planned on short-axis pilots at 18° angles to each other to visualize all 17 segments) and analyzed with dedicated software (Circle, cvi42, Calgary, AB, Canada; version 5.11.4).

Tissue tracking (TT) analysis was also performed to obtain strain data. TT analysis was performed on resting cine imagines. LVOT and mitral valve planes were excluded from the analysis.

Global 2 d longitudinal (GLS), circumferential (GCS), and radial (GRS) strain values were recorded.

Standard stress protocol included infusion of 140–210 mg/kg/min of adenosine (heart rate increase at peak stress >10% above baseline), for up to 6 min. First-pass perfusion data were acquired after injection of Gadobutrol 0.05 mmol/kg (Gadovist^®^, Bayer AG, Zurich, Switzerland) at 5 mL/s, followed by a 15-mL saline in 3 short axis slices using a breath-hold T1-weighted fast gradient echo sequence. Beta-blocker drugs were stopped five days prior to examination while nitrates, calcium-channel blockers and ACE inhibitors were interrupted two days before, as for caffeine or theine.

Ischemia was defined as a sub-endocardial hypointense area in the left ventricle wall during first-pass perfusion, evident in at least three frames beyond peak contrast enhancement. Significant cut-off considered was two or more neighboring segments, two adjacent slices, or a single transmural segment (approximately 6% of the myocardium). Extension of ischemia (ischemic burden) was defined as the sum of involved segments.

LGE sequences were analyzed to exclude from the analysis all patients with myocardial scarring indicative of previous MI.

### 2.2. Study Outcomes and Patient’s Follow-Up

Follow-up time was considered as the time lapse from the last examination to the interview. Primary outcomes were HF and all major cardiac events (MACE) including death from cardiovascular causes, myocardial infarction, hospitalization for unstable angina, or resuscitated cardiac arrest. Secondary outcomes were HF.

### 2.3. Statistical Analysis

Descriptive variables are presented as average and correspondent confidential intervals or as percentages (frequencies). The Shapiro–Wilk (SW) test was used to evaluate data distribution. A t-test was used for normal variables comparison; a chi-squared test was used with nominal (dichotomic) variables. The healthy group was used to define normal strain values. A comparison of strain data between long-standing CCS patients and healthy participants was performed. The probability of having primary outcomes given variables was estimated as odds/1 + odds. These analyses were performed with SPSS (IBM Corp. Released 2016. IBM SPSS Statistics for Mac, Version 26.0. Armonk, NY, USA: IBM Corp.). The outcome has been modelled performing exact logic regression to account for the small sample size. Model fitting has been assessed using the probability score of each model. Model diagnostic performance has been addressed carrying out a ROC analysis. Exact logistic regression was performed via Stata (StataCorp. 2021. Stata Statistical Software: Release 17. College Station, TX, USA: StataCorp LLC.). This analysis was also re-tested with a surrogate test via NCSS 2022 Statistical Software (2022, NCSS, LLC., Kaysville, UT, USA), which confirmed the same results. A non-inferiority test (with 0.1 margin) for two AUCs built up using the models described above was performed via NCSS 2022 Statistical Software. An alpha error of 5% was used as a threshold of significance.

## 3. Results

### 3.1. Patient Characteristics

The study population consisted of 35 patients (29 M, 6 F, mean age of 69 ± 9 years) referred for long-standing CCS, all managed with MT at the time of the scan. Mean follow-up was 3.73 years (interquartile range: from 1 to 6).

No major nor minor complication occurred during spCMR.

The baseline characteristics of study participants are listed in Table 1.

Perfusion images and LGE were considered of good quality in all cases examined.

Mean ejection fraction was 61% ± 8%, meaning an overall preserved systolic function of the study participants.

Twelve patients (31%) referred primary outcomes, seven of which (20%) with heart failure syndrome, and one died (3%).

### 3.2. Chronic Coronary Syndrome Characteristics

Twenty-one patients (60%) showed a multivessel disease and were more likely symptomatic, referring typical chest pain or dyspnea (13 out of 16 symptomatic patients).

Ischemia was detected in 12 patients. No LGE was included in the analysis.

Multivessel disease was significantly associated with ischemia (10 out of 12 ischemic patients: *p*-value 0.045).

In patients with ischemia, mean ischemic burden (expressed as a percentage) was 9% ± 3%.

All strain values were lower when compared with healthy group (GLS: −16 ± 2 vs. −18 ± 1, *p*-value: 0.001; GCS: −17 ± 3 vs. −20 ± 2, *p*-value: 0.0001; GRS: 28 ± 7 vs. 36 ± 6, *p*-value: 0.0001).

**Figure 1:**
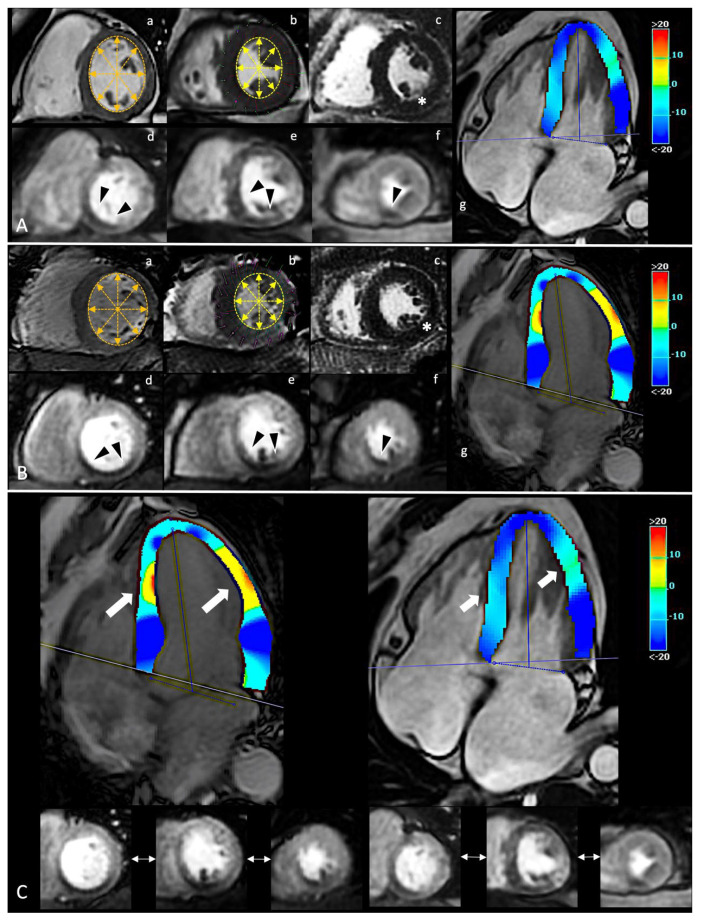
In panel (**A**,**B**), spCMR findings of two CCS patients. In (a,b), a single frame from SA cine sequence in diastolic and systolic phase, respectively; changes of inner double-arrows lines length highlight preserved systolic contraction. In (c), evidence of no enhancement in LGE sequences (white asterisk). In (d–f), a single frame from first-pass perfusion during adenosine infusion with evidence of similar ischemic burden involved inferior segments from the base to the apex (black arrowheads). In (g), a single systolic frame from HLA cine sequence with superimposed colorimetric map of GLS. On the right, the legend of colorimetric map with correlation between values and colors. In panel (**C**), the comparison between the different GLS. Despite a similar ischemic burden, the two patients report different GLS abnormalities (white thick arrows), suggesting a different impact of ischemia on global deformability. GLS acts as accurate index of early global impairment beyond the focal injury. spCMR: stress perfusion cardiac magnetic resonance; CCS: chronic coronary syndrome; SA: short-axis; LGE: late gadolinium enhancement; HLA: horizontal long-axis; GLS: global longitudinal strain.

However, the GLS only resulted significantly different for CCS patients categorized for the presence of ischemia (−14 ± 2% vs. −17 ± 1%, *p*-value 0.0001) (Figure 1).

No significant difference was detected between ischemic patients for other strain values.

Ischemic burden correlates with GLS (r: 0.699, *p*-value: 0.0001). Moreover, when GLS was categorized for reduced or preserved values, ischemic burden was predictive of GLS impairment (OR: 1.33, 95%CI: 1.08–1.64; *p*-value 0.008).

Among patients with multivessel diseases, patients with a three-vessel disease (TVD) were more likely associated also with all-strain anomalies (4 out of 5 patients with all-strain anomalies showed a TVD; *p*-value 0.007).

### 3.3. Association with Outcomes

Among all variables, multivessel diseases, symptoms, ischemia, and GLS involvement were associated with primary outcomes (Table 2). Probability to experience primary outcomes based on symptoms was 62% and increase to 67% when multivessel disease was also considered. 

Probability to experience primary outcomes when ischemia only was detected was 75% and increased to 91% when multivessel disease and symptoms also were considered.

Higher burden of ischemia was predictive of disease progression (i.e., occurrence of primary or secondary outcomes) (OR: 1.59, 95% CI: 1.18–2.14; *p*-value 0.002).

A predictive model including all multivessel diseases, symptoms, ischemia, and strain anomalies (Model I) reached an AUC of 0.93 (95% CI: 0.83–1). 

Model II (including only ischemia and strain anomalies) reached an AUC of 0.89 (95% CI: 0.74–0.97).

Lastly, Model III (including only symptoms and multivessel diseases) reached an AUC of 0.82 (95% CI: 0.67–0.97).

**Figure 2:**
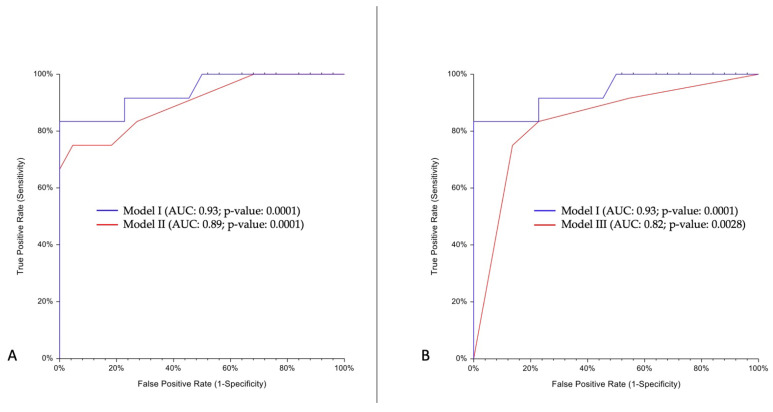
Non-inferiority test for two AUCs. Model I (symptoms, multivessel diseases, ischemia, and strain anomalies); Model II (ischemia and strain anomalies); Model III (symptoms and multivessel diseases). Panel (**A**) shows comparison between Model I (blue line) and II (red line) AUCs. In panel (**B**), comparison between Model I (blue line) and Model III (red line) AUCs. AUC: area under curve.

Using Model I as reference, Model II was non-inferior to model I (AUC difference: −0.04; One-Sided 95% lower limit: −0.09; Non-Inferiority *p*-value: 0.033). Conversely, the non-inferiority test failed for Model III (AUC difference: −0.1; One-Sided 95% lower limit: −0.22; Non-Inferiority *p*-value: 0.524) (Figure 2).

## 4. Discussion

This study is a historical prospective analysis of patients referred to our institution for a long-standing CCS, managed with MT and evidence of preserved systolic function and no previous MI, consecutively undergoing both CCTA and spCMR over a short period for a comprehensive evaluation.

In 100% of cases, it was possible to carry out a clinical follow-up, including both clinical evaluations obtained through an on-site interview and all information collected through RIS for any hospitalizations.

Our analysis highlights some important findings:

(i) stratification considering symptoms, anatomical extension of disease, and ischemia showed significant association with primary outcomes including all composite MACE;

(ii) extensive disease was more likely associated with ischemia, and when extensive disease and ischemia were considered, high probability of early alterations of myocardial deformability were also detected in patients with preserved EF; and

(iii) inducible ischemia and early alteration of myocardial deformability showed a non-inferior ability to predict clinical evolution of long-standing CCS compared to the model also including symptoms and anatomical extension of disease.

Long-standing CCS affects a highly heterogeneous population in need of complex therapies, and management of these “complex” patients is a tricky process, which historically has considered several factors other than ischemia [34].

The main decision-making point was in fact based on anatomic level of lesions, symptoms, and clinical conditions.

Other conventional imaging outcome predictors include LVEF, more recently echocardiographic GLS, and LGE, with LVEF considered as a major predictor of long-term survival in patients with CAD.

In latest years, cardiac imaging has shown high accuracy in guiding standard-of-care coronary revascularization, although the recent ISCHEMIA trial (or the controverse COURAGE or BARI-2D) failed to show a real advantage in using an early invasive revascularization in patients stratified for moderate-to-severe ischemia [14,15,35,36]. Moreover, in a sub study of ISCHEMIA by Reynolds et al., extent of ischemia is also a poor discriminator of risk for most clinical end-points [13,14,15].

It is therefore legitimate to question the role of imaging findings in a real-world scenario of these complex patients, especially when conventional outcome predictors are missing [37,38].

ISCHEMIA reveals that 70% of all ischemia-tests performed were perfusion imaging tests and that the predominant tool was nuclear perfusion imaging. To confirm this, spCMR continues to be an underutilized tool, accounting for <0.1% of all tests used in 2018, according to US statistics [39].

However, other focused trials and large registry studies have shown that spCMR had a high clinical impact in stratification of ischemic patients [16,19,20,21,22,23,40,41].

Regarding this strongest evidence, recent AHA guidelines for the evaluation and diagnosis of chest pain emphasize the primary role of spCMR in the identification of myocardial ischemia for a proper management of patients with acute chest pain and no known CAD [42].

In MR-INFORM RCT, spCMR-related ischemia proved similar to FFR in stratifying ischemic patients with no significant difference in MACE occurrence between spCMR and FFR-harm [43].

In SPINS registry, similarly to other studies, extensive ischemic burden was related to a higher risk of long-term, all-cause mortality, and revascularization was associated with a protective effect only in the restricted subset of patients with extensive spCMR-related ischemia [44,45,46].

Similarly, our results showed that quantification of ischemic burden improved the prognostication of CCS patients.

Moreover, as shown by Ge et al. through the same SPINS registry, spCMR did not suffer from the same limitation of CT (i.e., elevated BMI did not negatively impact its diagnostic quality) [47]. In our case series, spCMR confirmed high diagnostic quality in all examinations, irrespective of age.

spCMR has several advantages to other perfusion techniques, allowing an in vivo modeling of the heart including perfusion, tissue characterization and systolic function. High attention is paid also on CMR strain which has shown relevant impact in several cardiomyopathies [48,49,50,51,52,53,54].

CMR-derived cardiac model could therefore represent an effective tool in guide IHD management.

### CCS and Outcome Association

During the latest years, CCTA strongly impact the management of heart disease both in routine and emergency settings [55,56,57,58,59,60,61,62,63,64,65,66,67]. Clinical trial also highlighted the impact of CAD definition from CCTA [68,69,70].

From PROMISE, CCTA detects CAD better than conventional stress testing (i.e., not including spCMR), and thus, with a better prediction of cardiac event, especially in non-obstructive CAD. In this regard, the analysis by Hoffmann et al. shows that the ability of CCTA to identify a low-risk group correspond to an event rate of 0.9% over a two-year period vs. 2.1% observed in patients managed with normal stress test [10,11,12].

Similarly, from SCOT-HEART, management of CAD patients based on CT findings is revealed to be effective vs. the standard-of-care only, also guiding the clinicians in adopting medical treatment to prevent major events [59,71].

Presence, extension, and severity showing advantages in stratifying CAD patients also in CONFIRM study vs. clinical scoring, e.g., Framingham or Morise scores [72].

Therefore, CCTA is primarily adopted in CAD rule-out, with a negative predictive value close to 100%, enhanced by the effective dose optimization protocols and current technologies improved though the use of artificial intelligence, but is limited in detecting ischemia, the effective myocardial injurer, unless functional markers as with FFR-CT or CT-MPI are adopted [73,74,75,76,77,78].

In our study, CAD anatomy proved effective in the definition of outcomes, with the CAD extension significantly associated with composite MACE. However, the current inability of standard analysis to provide functional information capable to predict the clinical evolution of longstanding CCS patients affect the overall accuracy of Model III, which resulted inferior to the reference (i.e., the model including all variables).

As highlighted in FAME(s) and similar studies, the most important prognostic factor of a given coronary artery stenosis with respect to cardiac death or MI is indeed its ability to produce myocardial ischemia [79,80,81,82,83,84].

Ischemia is also the main predictor of HF evolution of CCS patients.

The pathophysiological process underlying the development of HF in ischemic patients can be variable and recognize different and specific therapeutic management [85,86].

In HFpEF models, recent evidence suggests that the onset of coronary microvascular dysfunctions (CMD) in non-infarcted areas contributes to the recurrence of ischemia which determines the progression of organ dysfunction as the onset of congestion symptoms even in absence of a real impairment of global systolic function [87,88,89,90,91].

Evidence suggests the potential occurrence of CMD in patients with CCS. In the CE-MARC 2 coronary physiology sub-study, a high incidence of CMD was found in patients with obstructive and non-obstructive CAD [92].

The identification of ischemic substrate in absence of obstructive CAD (more likely due to microvascular injury) is of primary importance considering that standardized approaches often fail in a correct assessment and management of CMD patients as is evident by the recent CorMicA trial [93,94,95,96,97,98].

Therefore, the identification of ischemia as a territory-specific assessment, irrespective of lesion-specific assessments, proves necessary for a proper treatment.

The following evidence, in concordance with our results, bring about some considerations:

1. spCMR findings result as good predictors of clinical evolution of CCS patients beyond symptoms and CAD extension. The probability of developing MACE was about 90% when ischemia was detected, with a high prevalence of HF syndrome during follow-up.
2. CMR-related strain confirms its ability to stage myocardial damage, which could translate into a critical ability to predict disease progression [99,100]. Among CCS patients with ischemia and no other conventional imaging predictor, GLS resulted highly impaired with a good correlation with the ischemic burden. This correlation proves GLS (an indicator of global function) as effective in describing the real impact of ischemia on cardiac function beyond the localized distribution of ischemic damage [101].
3. Despite GLS significantly differing between ischemic and non-ischemic CCS patients, GCS and GRS results were impaired when compared to a healthy population. Actually, in our series, GCS showed a stable early impairment compared to healthy volunteers. GCS impairment is indeed more likely related to a transmural injury/advanced disease, while GLS resulted most sensitive to a subendocardial/early injury [99]. On the other hand, the lack of a significant difference of GRS between ischemic and non-ischemic longstanding CCS patients could be explained by a relatively preserved compensating mechanism offered by circumferential fibers, since radial strain is tethered with other longitudinal and circumferential fibers and no radially oriented fibers are disposed within the myocardium.

This study presents some obvious limits. (i) This study is based on a retrospective analysis of a small study sample with the consequent risk of a potential selection bias, although all patients meeting the inclusion criteria were included in the prospective follow-up, uncensored real world picture of patients with longstanding CCS; (ii) this is a single-center study, although the single-center reference allowed for the obtaining of all the clinical information also included in the RIS and the standardization of the approach to patients and clinical information; (iii) although the individual therapeutic schemes were included in the interview, we are not able to identify all cardiological therapeutic modifications based on CCTA and stress CMR findings; thus, therapeutic modifications that did not include an invasive approach were not considered for the overall clinical evolution; (iv) CMR-TT analysis was performed on cine images acquired only during rest condition (i.e., not with increased strain values), and therefore it is not possible to conclude about strain anomalies during stress condition.

## 5. Conclusions

In vivo spCMR-modeling including perfusion and strain anomalies could represent a powerful tool in long-standing CCS, even when conventional imaging predictors are missing. In addition to the definition of death and MI risk, spCMR-modeling could also predict the clinical evolution trends toward HF as in our series of long-standing CCS patients with preserved systolic function and no previous MI, thus identifying patients who deserve more aggressive treatment, although larger studies are needed to fully clarify this issue.

## Figures and Tables

**Table 1:** Baseline patient characteristics.

		PO (No)	PO (Yes)	*p*-Value
All *n* (%)	35 (100)	23 (66)	12 (34)	
Sex (male) *n* (%)	29 (83)	21 (60)	8 (23)	0.089
Sex (female) *n* (%)	6 (17)	2 (6)	4 (11)	
Age (years)	69 ± 9	67 ± 9	72 ± 7	0.122
EF (%)	61 ± 8	60 ± 7	63 ± 10	0.415
Diabetes *n* (%)	6 (17)	3 (9)	3 (9)	0.329
Hypertension *n* (%)	13 (37)	9 (26)	4 (11)	0.517
Smoking habits *n* (%)	8 (23)	6 (17)	2 (6)	0.429
Familiarity for CHD *n* (%)	10 (29)	8 (23)	2 (6)	0.236
Dyslipidemia *n* (%)	19 (54)	12 (34)	7 (20)	0.505
Symptoms *n* (%)	16 (46)	6 (17)	10 (29)	0.002 **
Multivessel CAD *n* (%)	21 (60)	11 (31)	10 (29)	0.045 *
Ischemia *n* (%)	12 (34)	3 (9)	9 (26)	0.0001 **
Ischemic burden (%)	9 ±3	1 ± 2	7 ± 5	0.0001 **
CMR-Tissue Tracking CCS group				
GLS (%)	−16 ± 2	−17 ± 1	−14 ± 2	0.0001 **
GCS (%)	−17 ± 3	−17 ± 2	−17 ± 4	0.489
GRS (%)	28 ± 7	27 ± 6	29 ± 10	0.147
CMR-Tissue Tracking Healthy group				
GLS (%)	−18 ± 1			
GCS (%)	−20 ± 2			
GRS (%)	36 ± 6			

PO: primary outcomes; EF: ejection fraction; CHD: coronary heart disease; CAD: coronary artery disease; CMR: cardiac magnetic resonance; GLS: global longitudinal strain; GCS: global circumferential strain; GRS: global radial strain. significant difference *: level of significance: *p*-value < 0.05; ** significant difference: level of significance: *p*-value < 0.01.

**Table 2:** Primary and Secondary Outcome According to Symptoms, Multivessel Disease, Ischemia and Strain.

	Symptoms	Multivessel Disease	Ischemia	GLS Impairment	Ischemia and GLS Impairment
No of patients	16 (46)	21 (60)	12 (34)	13 (37)	8 (23)
MACE *n* (%)	10 (63)	10 (48)	9 (75)	9 (69)	8 (100)
HF *n* (%)	7 (44)	6 (29)	6 (50)	5 (38)	5 (63)
no MACE or HF *n* (%)	6 (37)	11 (52)	3 (25)	4 (31)	0

MACE: major adverse cardiac events; HF: heart failure.

## Data Availability

Data available to request.

## References

[1] Knuuti J., Wijns W., Saraste A., Capodanno D., Barbato E., Funck-Brentano C., Prescott E., Storey R.F., Deaton C., Cuisset T. (2020). 2019 ESC Guidelines for the diagnosis and management of chronic coronary syndromes: The Task Force for the diagnosis and management of chronic coronary syndromes of the European Society of Cardiology (ESC). Eur. Heart J..

[2] Danad I., Szymonifka J., Twisk J.W., Nørgaard B., Zarins C.K., Knaapen P., Min J.K. (2016). Diagnostic performance of cardiac imaging methods to diagnose ischaemia-causing coronary artery disease when directly compared with fractional flow reserve as a reference standard: A meta-analysis. Eur. Heart J..

[3] Esposito A., Gallone G., Palmisano A., Marchitelli L., Catapano F., Francone M. (2020). The current landscape of imaging recommendations in cardiovascular clinical guidelines: Toward an imaging-guided precision medicine. Radiol. Med..

[4] Ciancarella P., Ciliberti P., Santangelo T.P., Secchi F., Stagnaro N., Secinaro A. (2020). Noninvasive imaging of congenital cardiovascular defects. Radiol. Med..

[5] La Grutta L., Toia P., Grassedonio E., Pasta S., Albano D., Agnello F., Maffei E., Cademartiri F., Bartolotta T.V., Galia M. (2020). TAVI imaging: Over the echocardiography. Radiol. Med..

[6] Takehara Y. (2020). 4D Flow when and how?. Radiol. Med..

[7] Palmisano A., Darvizeh F., Cundari G., Rovere G., Ferrandino G., Nicoletti V., Cilia F., De Vizio S., Palumbo R., Esposito A. (2021). Advanced cardiac imaging in athlete’s heart: Unravelling the grey zone between physiologic adaptation and pathology. Radiol. Med..

[8] Schicchi N., Fogante M., Palumbo P., Agliata G., Pirani P.E., Di Cesare E., Giovagnoni A. (2020). The sub-millisievert era in CTCA: The technical basis of the new radiation dose approach. Radiol. Med..

[9] Ledda R.E., Milanese G., Cademartiri F., Maffei E., Benedetti G., Goldoni M., Silva M., Sverzellati N. (2021). Association of hepatic steatosis with epicardial fat volume and coronary artery disease in symptomatic patients. Radiol. Med..

[10] Hoffmann U., Truong Q.A., Schoenfeld D.A., Chou E.T., Woodard P.K., Nagurney J.T., Pope J.H., Hauser T.H., White C.S., Weiner S. (2012). Coronary CT Angiography versus Standard Evaluation in Acute Chest Pain. N. Engl. J. Med..

[11] Douglas P.S., Hoffmann U., Patel M.R., Mark D.B., Al-Khalidi H.R., Cavanaugh B., Cole J., Dolor R., Fordyce C.B., Huang M. (2015). Outcomes of Anatomical versus Functional Testing for Coronary Artery Disease. N. Engl. J. Med..

[12] Hoffmann U., Ferencik M., Udelson J.E., Picard M.H., Truong Q.A., Patel M.R., Huang M., Pencina M., Mark D.B., Heitner J.F. (2017). Prognostic Value of Noninvasive Cardiovascular Testing in Patients With Stable Chest Pain. Circulation.

[13] Newby D.E., Williams M.C., Dweck M.R. (2021). Forget Ischemia: It’s All About the Plaque. Circulation.

[14] Reynolds H.R., Picard M.H., Spertus J.A., Peteiro J., Sendon J.L.L., Senior R., El-Hajjar M.C., Celutkiene J., Shapiro M.D., Pellikka P.A. (2021). Natural History of Patients With Ischemia and No Obstructive Coronary Artery Disease. Circulation.

[15] Reynolds H.R., Shaw L.J., Min J.K., Page C.B., Berman D.S., Chaitman B.R., Picard M.H., Kwong R.Y., O’Brien S.M., Huang Z. (2021). Outcomes in the ISCHEMIA Trial Based on Coronary Artery Disease and Ischemia Severity. Circulation.

[16] Pezel T., Silva L.M., Bau A.A., Teixiera A., Jerosch-Herold M., Coelho-Filho O.R. (2021). What Is the Clinical Impact of Stress CMR After the ISCHEMIA Trial?. Front. Cardiovasc. Med..

[17] Velazquez E.J., Lee K.L., Jones R.H., Al-Khalidi H.R., Hill J.A., Panza J.A., Michler R.E., Bonow R.O., Doenst T., Petrie M.C. (2016). Coronary-Artery Bypass Surgery in Patients with Ischemic Cardiomyopathy. N. Engl. J. Med..

[18] Ge Y., Antiochos P., Steel K., Bingham S., Abdullah S., Chen Y.-Y., Mikolich J.R., Arai A.E., Bandettini W.P., Shanbhag S.M. (2020). Prognostic Value of Stress CMR Perfusion Imaging in Patients With Reduced Left Ventricular Function. JACC Cardiovasc. Imaging.

[19] Patel A.R., Salerno M., Kwong R.Y., Singh A., Heydari B., Kramer C.M. (2021). Stress Cardiac Magnetic Resonance Myocardial Perfusion Imaging. J. Am. Coll. Cardiol..

[20] Pezel T., Garot P., Hovasse T., Unterseeh T., Champagne S., Kinnel M., Toupin S., Louvard Y., Morice M.C., Sanguineti F. (2021). Vasodilatation stress cardiovascular magnetic resonance imaging: Feasibility, workflow and safety in a large prospective registry of more than 35,000 patients. Arch. Cardiovasc. Dis..

[21] Pezel T., Unterseeh T., Garot P., Hovasse T., Kinnel M., Champagne S., Toupin S., Sanguineti F., Garot J. (2021). Prognostic value of vasodilator stress perfusion cardiovascular magnetic resonance after inconclusive stress testing. J. Cardiovasc. Magn. Reson..

[22] Pezel T., Unterseeh T., Garot P., Hovasse T., Sanguineti F., Toupin S., Morisset S., Champagne S., Garot J. (2021). Long-Term Prognostic Value of Stress Cardiovascular Magnetic Resonance–Related Coronary Revascularization to Predict Death: A Large Registry With >200,000 Patient-Years of Follow-Up. Circ. Cardiovasc. Imaging.

[23] Pavon A.G., Porretta A.P., Arangalage D., Domenichini G., Rutz T., Hugelshofer S., Pruvot E., Monney P., Pascale P., Schwitter J. (2022). Feasibility of adenosine stress cardiovascular magnetic resonance perfusion imaging in patients with MR-conditional transvenous permanent pacemakers and defibrillators. J. Cardiovasc. Magn. Reson..

[24] Lipinski M.J., McVey C.M., Berger J., Kramer C.M., Salerno M. (2013). Prognostic Value of Stress Cardiac Magnetic Resonance Imaging in Patients With Known or Suspected Coronary Artery Disease: A Systematic Review and Meta-Analysis. J. Am. Coll. Cardiol..

[25] Centonze M., Steidler S., Casagranda G., Alfonsi U., Spagnolli F., Rozzanigo U., Palumbo D., Faletti R., De Cobelli F. (2020). Cardiac-CT and cardiac-MR cost-effectiveness: A literature review. Radiol. Med..

[26] Buffa V., Di Renzi P. (2020). CMR in the diagnosis of ischemic heart disease. Radiol. Med..

[27] Desai R.R., Jha S. (2013). Diagnostic Performance of Cardiac Stress Perfusion MRI in the Detection of Coronary Artery Disease Using Fractional Flow Reserve as the Reference Standard: A Meta-Analysis. Am. J. Roentgenol..

[28] Leiner T., Bogaert J., Friedrich M.G., Mohiaddin R., Muthurangu V., Myerson S., Powell A.J., Raman S.V., Pennell D.J. (2020). SCMR Position Paper (2020) on clinical indications for cardiovascular magnetic resonance. J. Cardiovasc. Magn. Reson..

[29] Klem I., Klein M., Khan M., Yang E.Y., Nabi F., Ivanov A., Bhatti L., Hayes B., Graviss E.A., Nguyen D.T. (2021). Relationship of LVEF and Myocardial Scar to Long-Term Mortality Risk and Mode of Death in Patients With Nonischemic Cardiomyopathy. Circulation.

[30] Hachamovitch R. (2013). Impact of ischemia and scar on therapeutic benefit of myocardial revascularization. Herz.

[31] Kwon D.H., Obuchowski N.A., Marwick T.H., Menon V., Griffin B., Flamm S.D., Hachamovitch R. (2018). Jeopardized Myocardium Defined by Late Gadolinium Enhancement Magnetic Resonance Imaging Predicts Survival in Patients With Ischemic Cardiomyopathy: Impact of Revascularization. J. Am. Heart Assoc..

[32] Craft J., Li Y., Bhatti S., Cao J.J. (2021). How to do left atrial late gadolinium enhancement: A review. Radiol. Med..

[33] Palmisano A., Vignale D., Benedetti G., Del Maschio A., De Cobelli F., Esposito A. (2020). Late iodine enhancement cardiac computed tomography for detection of myocardial scars: Impact of experience in the clinical practice. Radiol. Med..

[34] Kip K.E., Hollabaugh K., Marroquin O.C., Williams D.O. (2008). The Problem With Composite End Points in Cardiovascular Studies: The Story of Major Adverse Cardiac Events and Percutaneous Coronary Intervention. J. Am. Coll. Cardiol..

[35] Boden W.E., O’Rourke R.A., Teo K.K., Hartigan P.M., Maron D.J., Kostuk W.J., Knudtson M., Dada M., Casperson P., Harris C.L. (2007). Optimal Medical Therapy with or without PCI for Stable Coronary Disease. N. Engl. J. Med..

[36] Frye R.L., August P., Brooks M.M., Hardison R.M., Kelsey S.F., MacGregor J.M., Orchard T.J., Chaitman B.R., Genuth S.M., BARI 2D Study Group (2009). A Randomized Trial of Therapies for Type 2 Diabetes and Coronary Artery Disease. N. Engl. J. Med..

[37] Xie J.X., Winchester D.E., Phillips L.M., Hachamovitch R., Berman D.S., Blankstein R., Di Carli M.F., Miller T.D., Al-Mallah M.H., Shaw L.J. (2017). The elusive role of myocardial perfusion imaging in stable ischemic heart disease: Is ISCHEMIA the answer?. J. Nucl. Cardiol..

[38] Mani P., Hachamovitch R. (2020). Can Stress Cardiac Magnetic Resonance Identify Potential Survival Benefit With Revascularization in Stable Ischemic Heart Disease?. JACC Cardiovasc. Imaging.

[39] Schwitter J. (2019). The SPINS Trial: Building Evidence and a Consequence?. J. Am. Coll. Cardiol..

[40] Hendel R.C., Friedrich M.G., Schulz-Menger J., Zemmrich C., Bengel F., Berman D.S., Camici P.G., Flamm S.D., Le Guludec D., Kim R. (2016). CMR First-Pass Perfusion for Suspected Inducible Myocardial Ischemia. JACC Cardiovasc. Imaging.

[41] Kwong R.Y., Ge Y., Steel K., Bingham S., Abdullah S., Fujikura K., Wang W., Pandya A., Chen Y.-Y., Mikolich J.R. (2019). Cardiac Magnetic Resonance Stress Perfusion Imaging for Evaluation of Patients With Chest Pain. J. Am. Coll. Cardiol..

[42] Gulati M., Levy P.D., Mukherjee D., Amsterdam E., Bhatt D.L., Birtcher K.K., Blankstein R., Boyd J., Bullock-Palmer R.P., Conejo T. (2021). 2021 AHA/ACC/ASE/CHEST/SAEM/SCCT/SCMR Guideline for the Evaluation and Diagnosis of Chest Pain: Executive Summary: A Report of the American College of Cardiology/American Heart Association Joint Committee on Clinical Practice Guidelines. Circulation.

[43] Nagel E., Greenwood J.P., McCann G.P., Bettencourt N., Shah A.M., Hussain S.T., Perera D., Plein S., Bucciarelli-Ducci C., Paul M. (2019). Magnetic Resonance Perfusion or Fractional Flow Reserve in Coronary Disease. N. Engl. J. Med..

[44] Farzaneh-Far A., Borges-Neto S. (2011). Ischemic Burden, Treatment Allocation, and Outcomes in Stable Coronary Artery Disease. Circ. Cardiovasc. Imaging.

[45] Hachamovitch R. (2015). Does Ischemia Burden in Stable Coronary Artery Disease Effectively Identify Revascularization Candidates?. Circ. Cardiovasc. Imaging.

[46] Marcos-Garces V., Gavara J., Monmeneu J.V., Lopez-Lereu M.P., Bosch M.J., Merlos P., Perez N., Rios-Navarro C., De Dios E., Bonanad C. (2020). Vasodilator Stress CMR and All-Cause Mortality in Stable Ischemic Heart Disease. JACC Cardiovasc. Imaging.

[47] Ge Y., Steel K., Antiochos P., Bingham S., Abdullah S., Mikolich J.R., Arai A.E., Bandettini W.P., Shanbhag S.M., Patel A.R. (2021). Stress CMR in patients with obesity: Insights from the Stress CMR Perfusion Imaging in the United States (SPINS) registry. Eur. Heart J. Cardiovasc. Imaging.

[48] Galea N., Polizzi G., Gatti M., Cundari G., Figuera M., Faletti R. (2020). Cardiovascular magnetic resonance (CMR) in restrictive cardiomyopathies. Radiol. Med..

[49] Liguori C., Farina D., Vaccher F., Ferrandino G., Bellini D., Carbone I. (2020). Myocarditis: Imaging up to date. Radiol. Med..

[50] Palumbo P., Cannizzaro E., Di Cesare A., Bruno F., Schicchi N., Giovagnoni A., Splendiani A., Barile A., Masciocchi C., Di Cesare E. (2020). Cardiac magnetic resonance in arrhythmogenic cardiomyopathies. Radiol. Med..

[51] Palumbo P., Masedu F., De Cataldo C., Cannizzaro E., Bruno F., Pradella S., Arrigoni F., Valenti M., Splendiani A., Barile A. (2021). Real-world clinical validity of cardiac magnetic resonance tissue tracking in primitive hypertrophic cardiomyopathy. Radiol. Med..

[52] Pierpaolo P., Rolf S., Manuel B.-P., Davide C., Dresselaers T., Claus P., Bogaert J. (2020). Left ventricular global myocardial strain assessment: Are CMR feature-tracking algorithms useful in the clinical setting?. Radiol. Med..

[53] Pradella S., Grazzini G., De Amicis C., Letteriello M., Acquafresca M., Miele V. (2020). Cardiac magnetic resonance in hypertrophic and dilated cardiomyopathies. Radiol. Med..

[54] Russo V., Lovato L., Ligabue G. (2020). Cardiac MRI: Technical basis. Radiol. Med..

[55] Hadamitzky M., Freissmuth B., Meyer T., Hein F., Kastrati A., Martinoff S., Schömig A., Hausleiter J. (2009). Prognostic Value of Coronary Computed Tomographic Angiography for Prediction of Cardiac Events in Patients With Suspected Coronary Artery Disease. JACC Cardiovasc. Imaging.

[56] Hadamitzky M., Distler R., Meyer T., Hein F., Kastrati A., Martinoff S., Schömig A., Hausleiter J. (2011). Prognostic Value of Coronary Computed Tomographic Angiography in Comparison With Calcium Scoring and Clinical Risk Scores. Circ. Cardiovasc. Imaging.

[57] Andreini D., Pontone G., Mushtaq S., Bartorelli A.L., Bertella E., Antonioli L., Formenti A., Cortinovis S., Veglia F., Annoni A. (2012). A Long-Term Prognostic Value of Coronary CT Angiography in Suspected Coronary Artery Disease. JACC Cardiovasc. Imaging.

[58] Motoyama S., Ito H., Sarai M., Kondo T., Kawai H., Nagahara Y., Harigaya H., Kan S., Anno H., Takahashi H. (2015). Plaque Characterization by Coronary Computed Tomography Angiography and the Likelihood of Acute Coronary Events in Mid-Term Follow-Up. J. Am. Coll. Cardiol..

[59] Nadjiri J., Hausleiter J., Jähnichen C., Will A., Hendrich E., Martinoff S., Hadamitzky M. (2016). Incremental prognostic value of quantitative plaque assessment in coronary CT angiography during 5 years of follow up. J. Cardiovasc. Comput. Tomogr..

[60] Marano R., Rovere G., Savino G., Flammia F.C., Carafa M.R.P., Steri L., Merlino B., Natale L. (2020). CCTA in the diagnosis of coronary artery disease. Radiol. Med..

[61] Valente T., Pignatiello M., Sica G., Bocchini G., Rea G., Cappabianca S., Scaglione M. (2020). Hemopericardium in the acute clinical setting: Are we ready for a tailored management approach on the basis of MDCT findings?. Radiol. Med..

[62] Şeker M. (2019). Prevalence and morphologic features of dual left anterior descending artery subtypes in coronary CT angiography. Radiol. Med..

[63] Rovere G., Meduri A., Savino G., Flammia F.C., Piccolo F.L., Carafa M.R.P., Larici A.R., Natale L., Merlino B., Marano R. (2021). Practical instructions for using drugs in CT and MR cardiac imaging. Radiol. Med..

[64] Palumbo P., Cannizzaro E., Bruno F., Schicchi N., Fogante M., Agostini A., De Donato M.C., De Cataldo C., Giovagnoni A., Barile A. (2020). Coronary artery disease (CAD) extension-derived risk stratification for asymptomatic diabetic patients: Usefulness of low-dose coronary computed tomography angiography (CCTA) in detecting high-risk profile patients. Radiol. Med..

[65] Esposito A., Francone M., Andreini D., Buffa V., Cademartiri F., Carbone I., Clemente A., Guaricci A.I., Guglielmo M., Indolfi C. (2021). SIRM—SIC appropriateness criteria for the use of Cardiac Computed Tomography. Part 1: Congenital heart diseases, primary prevention, risk assessment before surgery, suspected CAD in symptomatic patients, plaque and epicardial adipose tissue characterization, and functional assessment of stenosis. Radiol. Med..

[66] De Rubeis G., Marchitelli L., Spano G., Catapano F., Cilia F., Galea N., Carbone I., Catalano C., Francone M. (2021). Radiological outpatient’ visits to avoid inappropriate cardiac CT examinations: An 8-year experience report. Radiol. Med..

[67] Pontone G., Di Cesare E., Castelletti S., De Cobelli F., De Lazzari M., Esposito A., Focardi M., Di Renzi P., Indolfi C., Lanzillo C. (2021). Appropriate use criteria for cardiovascular magnetic resonance imaging (CMR): SIC—SIRM position paper part 1 (ischemic and congenital heart diseases, cardio-oncology, cardiac masses and heart transplant). Radiol. Med..

[68] Motoyama S., Sarai M., Narula J., Ozaki Y. (2013). Coronary CT angiography and high-risk plaque morphology. Cardiovasc. Interv. Ther..

[69] Pontone G., Andreini D., Bartorelli A.L., Bertella E., Cortinovis S., Mushtaq S., Foti C., Annoni A., Formenti A., Baggiano A. (2013). A Long-Term Prognostic Value of CT Angiography and Exercise ECG in Patients with Suspected CAD. JACC Cardiovasc. Imaging.

[70] Seitun S., Clemente A., Maffei E., Toia P., La Grutta L., Cademartiri F. (2020). Prognostic value of cardiac CT. Radiol. Med..

[71] Nakanishi R., Osawa K., Kurata A., Miyoshi T. (2021). Role of coronary computed tomography angiography (CTA) post the ISCHEMIA trial: Precision prevention based on coronary CTA-derived coronary atherosclerosis. J. Cardiol..

[72] Van Rosendael A.R., Bax A.M., van den Hoogen I.J., Smit J.M., Al’Aref S.J., Achenbach S., Al-Mallah M.H., Andreini D., Berman D.S., Budoff M.J. (2020). Associations between dyspnoea, coronary atherosclerosis, and cardiovascular outcomes: Results from the long-term follow-up CONFIRM registry. Eur. Heart J. Cardiovasc. Imaging.

[73] Schicchi N., Mari A., Fogante M., Pirani P.E., Agliata G., Tosi N., Palumbo P., Cannizzaro E., Bruno F., Splendiani A. (2020). In vivo radiation dosimetry and image quality of turbo-flash and retrospective dual-source CT coronary angiography. Radiol. Med..

[74] Van Assen M., Muscogiuri G., Caruso D., Lee S.J., Laghi A., De Cecco C.N. (2020). Artificial intelligence in cardiac radiology. Radiol. Med..

[75] Scapicchio C., Gabelloni M., Barucci A., Cioni D., Saba L., Neri E. (2021). A deep look into radiomics. Radiol. Med..

[76] Nardone V., Reginelli A., Grassi R., Boldrini L., Vacca G., D’Ippolito E., Annunziata S., Farchione A., Belfiore M.P., Desideri I. (2021). Delta radiomics: A systematic review. Radiol. Med..

[77] Coppola F., Faggioni L., Regge D., Giovagnoni A., Golfieri R., Bibbolino C., Miele V., Neri E., Grassi R. (2021). Artificial intelligence: Radiologists’ expectations and opinions gleaned from a nationwide online survey. Radiol. Med..

[78] Cicero G., Ascenti G., Albrecht M.H., Blandino A., Cavallaro M., D’Angelo T., Carerj M.L., Vogl T.J., Mazziotti S. (2020). Extra-abdominal dual-energy CT applications: A comprehensive overview. Radiol. Med..

[79] Tonino P.A.L., De Bruyne B., Pijls N.H.J., Siebert U., Ikeno F., van’t Veer M., Klauss V., Manoharan G., Engstrøm T., Oldroyd K.G. (2009). Fractional flow reserve versus angiography for guiding percutaneous coronary intervention. N. Engl. J. Med..

[80] Pijls N.H., Fearon W.F., Tonino P.A., Siebert U., Ikeno F., Bornschein B., Veer M.V., Klauss V., Manoharan G., Engstrøm T. (2010). Fractional Flow Reserve Versus Angiography for Guiding Percutaneous Coronary Intervention in Patients With Multivessel Coronary Artery Disease: 2-Year Follow-Up of the FAME (Fractional Flow Reserve Versus Angiography for Multivessel Evaluation) Study. J. Am. Coll. Cardiol..

[81] De Bruyne B., Pijls N.H., Kalesan B., Barbato E., Tonino P.A., Piroth Z., Jagic N., Mobius-Winckler S., Rioufol G., Witt N. (2012). Fractional Flow Reserve–Guided PCI versus Medical Therapy in Stable Coronary Disease. N. Engl. J. Med..

[82] Patel M.R., Jeremias A., Maehara A., Matsumura M., Zhang Z., Schneider J., Tang K., Talwar S., Marques K., Shammas N.W. (2022). 1-Year Outcomes of Blinded Physiological Assessment of Residual Ischemia After Successful PCI. JACC Cardiovasc. Interv..

[83] Zhang D., Lv S., Song X., Yuan F., Xu F., Zhang M., Yan S., Cao X. (2015). Fractional flow reserve versus angiography for guiding percutaneous coronary intervention: A meta-analysis. Heart.

[84] Xaplanteris P., Fournier S., Pijls N.H., Fearon W.F., Barbato E., Tonino P.A., Engstrøm T., Kääb S., Dambrink J.-H., Rioufol G. (2018). Five-Year Outcomes with PCI Guided by Fractional Flow Reserve. N. Engl. J. Med..

[85] Elgendy I.Y., Mahtta D., Pepine C.J. (2019). Medical Therapy for Heart Failure Caused by Ischemic Heart Disease. Circ. Res..

[86] Di Cesare E., Carerj S., Palmisano A., Carerj M.L., Catapano F., Vignale D., Di Cesare A., Milanese G., Sverzellati N., Francone M. (2021). Multimodality imaging in chronic heart failure. Radiol. Med..

[87] Masi S., Rizzoni D., Taddei S., Widmer R.J., Montezano A.C., Lüscher T.F., Schiffrin E.L., Touyz R.M., Paneni F., Lerman A. (2021). Assessment and pathophysiology of microvascular disease: Recent progress and clinical implications. Eur. Heart J..

[88] Crea F., Camici P.G., Merz C.N.B. (2014). Coronary microvascular dysfunction: An update. Eur. Heart J..

[89] Taqueti V.R., Solomon S.D., Shah A.M., Desai A.S., Groarke J.D., Osborne M., Hainer J., Bibbo C.F., Dorbala S., Blankstein R. (2017). Coronary microvascular dysfunction and future risk of heart failure with preserved ejection fraction. Eur. Heart J..

[90] Sechtem U., Brown D.L., Godo S., Lanza G.A., Shimokawa H., Sidik N. (2020). Coronary microvascular dysfunction in stable ischaemic heart disease (non-obstructive coronary artery disease and obstructive coronary artery disease). Cardiovasc. Res..

[91] Padro T., Manfrini O., Bugiardini R., Canty J., Cenko E., De Luca G., Duncker D.J., Eringa E.C., Koller A., Tousoulis D. (2020). ESC Working Group on Coronary Pathophysiology and Microcirculation position paper on ‘coronary microvascular dysfunction in cardiovascular disease’. Cardiovasc. Res..

[92] Corcoran D., Young R., Adlam D., McConnachie A., Mangion K., Ripley D., Cairns D., Brown J., Bucciarelli-Ducci C., Baumbach A. (2018). Coronary microvascular dysfunction in patients with stable coronary artery disease: The CE-MARC 2 coronary physiology sub-study. Int. J. Cardiol..

[93] Ford T., Stanley B., Good R., Rocchiccioli P., McEntegart M., Watkins S., Eteiba H., Shaukat A., Lindsay M., Robertson K. (2018). Stratified Medical Therapy Using Invasive Coronary Function Testing in Angina. J. Am. Coll. Cardiol..

[94] Ford T., Berry C. (2019). How to Diagnose and Manage Angina Without Obstructive Coronary Artery Disease: Lessons from the British Heart Foundation CorMicA Trial. Interv. Cardiol. Rev. Res. Resour..

[95] Ford T., Ong P., Sechtem U., Beltrame J., Camici P.G., Crea F., Kaski J.-C., Merz C.N.B., Pepine C.J., Shimokawa H. (2020). Assessment of Vascular Dysfunction in Patients Without Obstructive Coronary Artery Disease. JACC Cardiovasc. Interv..

[96] Ford T.J., Stanley B., Sidik N., Good R., Rocchiccioli P., McEntegart M., Watkins S., Eteiba H., Shaukat A., Lindsay M. (2020). 1-Year Outcomes of Angina Management Guided by Invasive Coronary Function Testing (CorMicA). JACC Cardiovasc. Interv..

[97] Ford T.J., Corcoran D., Berry C. (2018). Stable coronary syndromes: Pathophysiology, diagnostic advances and therapeutic need. Heart.

[98] Ford T.J., Corcoran D., Oldroyd K.G., McEntegart M., Rocchiccioli P., Watkins S., Brooksbank K., Padmanabhan S., Sattar N., Briggs A. (2018). Rationale and design of the British Heart Foundation (BHF) Coronary Microvascular Angina (CorMicA) stratified medicine clinical trial. Am. Heart J..

[99] Geyer H., Caracciolo G., Abe H., Wilansky S., Carerj S., Gentile F., Nesser H.-J., Khandheria B., Narula J., Sengupta P.P. (2010). Assessment of Myocardial Mechanics Using Speckle Tracking Echocardiography: Fundamentals and Clinical Applications. J. Am. Soc. Echocardiogr..

[100] Voigt J.-U., Cvijic M. (2019). 2- and 3-Dimensional Myocardial Strain in Cardiac Health and Disease. JACC Cardiovasc. Imaging.

[101] Holmes A.A., Romero J., Levsky J.M., Haramati L.B., Phuong N., Rezai-Gharai L., Cohen S., Restrepo L., Ruiz-Guerrero L., Fisher J.D. (2017). Circumferential strain acquired by CMR early after acute myocardial infarction adds incremental predictive value to late gadolinium enhancement imaging to predict late myocardial remodeling and subsequent risk of sudden cardiac death. J. Interv. Card. Electrophysiol..

